# Evaluation of Printability, Color Difference, Translucency, and Surface Roughness over Time in a 3D-Printed TiO_2_-Containing Denture Base Resin: A Pilot Study

**DOI:** 10.3390/ma18153683

**Published:** 2025-08-05

**Authors:** Gregory Bennett, Mark W. Beatty, Bobby Simetich

**Affiliations:** Department of Adult Restorative Dentistry, College of Dentistry, University of Nebraska Medical Center, Lincoln, NE 68583, USA; mbeatty@unmc.edu (M.W.B.); bsimetic@unmc.edu (B.S.)

**Keywords:** 3D-printing, titanium nanoparticles, denture base resins, surface roughness, color difference, stereolithography

## Abstract

Recent evidence suggests that nano-TiO_2_ particles improve antimicrobial and physical properties when incorporated into dental prosthetic materials. However, there exists a paucity of information regarding their impact on material properties when the prosthetic materials are 3D-printed over time. The purpose of this study was to evaluate the time-dependent printability and surface property changes occurring in a 3D-printed denture base resin containing nano-titanium dioxide (TiO_2_) particles. A 0.4 wt% concentration of 30 nm rutile TiO_2_ nanoparticles was ultrasonically dispersed into a denture base resin. Disks were printed weekly using a Form 2 SLA printer until printing failed. Printability, surface roughness (R_a_), color difference (ΔE_ab_), and translucency parameters were measured across timepoints. Surface roughness was assessed via profilometry, while color and translucency were evaluated using a spectrophotometer under standardized conditions. Print failure occurred at week 8, beyond which the resin could no longer reliably produce full specimens. R_a_ roughness decreased from 3.83 µm to 0.48 µm, which denoted a significant time-dependent decrease (ρ = −0.733, *p* = 0.016). Color difference with the unmodified control declined from 26.32 to 17.13 ΔE_ab_ units (ρ = −0.976, *p* < 0.001). All printed samples exceeded the clinically acceptable thresholds for both R_a_ (0.2 µm) and ΔE_ab_ (<3.7). Although the printability of the resin–TiO2 mixture was maintained for 7 weeks, mixture homogeneity declined over time. TiO_2_ additions to a denture polymer produced significant changes in surface roughness and color that were not clinically acceptable. Results from this study illustrate the time dependence required for retaining surface properties in 3D-printed dentures containing nano-TiO_2_.

## 1. Introduction

More than 36 million North Americans live without teeth [1], and approximately 90 percent of them rely on dentures to provide esthetics and function on a daily basis. A recent publication suggested that by 2030, over 19% of China’s population will exceed 60 years of age, thereby leading to a substantial economic burden caused by tooth loss [2]. Edentulism has been associated with a wide range of conditions, such as type 2 diabetes, cardiovascular disease, and sleep disordered breathing [3]. The economic burden that underlies denture construction can be minimized through 3D printing, as it is more time efficient and less costly than traditional methods, while achieving similar levels of patient satisfaction [4,5].

Dental prostheses are exposed to a high microbial load, as up to 70% of denture wearers experience fungal overgrowth caused by Candida species [6]. This leads to denture stomatitis [7,8]. Numerous attempts towards developing an antimicrobial denture base material have been reported using traditional fabrication methods [9,10,11,12,13,14,15] as well as 3D printing [16,17,18,19,20,21,22,23,24]. Although results from various studies have shown initial success, the effectiveness of incorporating antimicrobial agents into denture base resins has not been demonstrated conclusively [14]. Various additives have been evaluated for improving mechanical and antimicrobial properties including metal nanoparticles (Ti, Zr, Ag) and quaternary ammonium compounds [17,18,20,21,22,23]. TiO_2_ nanoparticles were chosen for this study due to their demonstrated biocompatibility and antimicrobial activity at 0.4% concentration [18,25].

Published research has either omitted reporting the mixing method [17,23], or engaged a variety of techniques that include mixing, stirring, ultrasonic dispersion, and a combination of methods [18,20,21,22,24]. The omission of mixing method may indicate that it is a proprietary process.

Considering the variety of mixing options available, it is important to know whether a mixed resin remains printable over time. This is a necessary feature for point-of-care printing by the dental professional. Therefore, the primary aim of this pilot study was to evaluate the time-dependent printability and surface property changes in a 3D-printed denture base resin containing TiO_2_ nanoparticles. The null hypothesis was that no significant difference in surface roughness or optical properties would occur across print cycles (α = 0.05).

## 2. Materials and Methods

This in vitro pilot study evaluated the effects of incorporating titanium dioxide (TiO_2_) nanoparticles on the surface roughness, color difference, and translucency of a 3D-printed denture base resin over time. The aim was to assess the stability of a hybrid resin formulation, stored without additional intervention, thereby mimicking the commercial point-of-care use of printed nanocomposite resins.

### 2.1. Preparation of TiO_2_-Modified Denture Base Resin

A 0.4 wt% mixture of 30 nm rutile titanium dioxide (TiO_2_) nanoparticles (US Research Nanomaterials, Houston, TX, USA) was prepared by dispersing the nanoparticles into a denture base resin (Denture Base Resin OP, Formlabs Inc., Somerville, MA, USA). Agglomerates were broken and particles dispersed using an ultrasonic processor equipped with a S3 sonotrode at amplitude 460 W/cm^2^ for 10 min (UP200S, Hielscher Ultrasonics GmbH, Teltow, Germany). An ice bath simultaneously cooled the mixture to prevent overheating of the monomer.

The TiO_2_-modified resin was loaded into a stereolithography (SLA) 3D printer equipped with a heated resin tank and mixing paddle (Form 2, Formlabs Inc., Somerville, MA, USA). A 35 mm diameter × 5 mm thickness disk design was created in Meshmixer (Autodesk Inc., San Rafael, CA, USA) and exported as an STL file for printing. Disks were printed flat on the build tray (0-degree orientation) with a layer height of 0.05 mm without support structures, with four specimens produced per print cycle.

Post-processing followed manufacturer-recommended protocols [26]. Printed specimens were washed in 91% isopropyl alcohol (IPA) for 30 min using a Form Wash unit (Formlabs Inc., Somerville, MA, USA), then air dried and post-cured in a glycerin bath at 60 °C for 60 min using a Form Cure unit at 405 nm wavelength (Formlabs Inc., Somerville, MA, USA).

Disks were printed with the unmodified resin to act as a control (T0) and then at multiple timepoints: immediately after mixing (T1), at day 3 (T2), and weekly thereafter until printing failure occurred (T3–T10). Failure was defined as the printer’s inability to fabricate all disks to full specifications. This occurred at week 8, when only one full disk and one incomplete (≤0.5 mm thickness) specimen were produced. Printed disks were stored dry in an airtight, dark container until testing. Due to incomplete printing at T10, only one disk was available per timepoint for analysis. In total, 11 disks (1 control and 10 experimental samples) were tested.

### 2.2. Surface Roughness Measurement

Surface roughness was measured using a Mitutoyo Surface Roughness Tester (model SJ-210, Mitutoyo Corp., Kanagawa, Japan). The instrument was placed on a flat, stable surface and connected to a power source. It was mounted to a stage using a custom fixture to prevent movement of both the instrument and the drive unit during testing.

Each disk (*n* = 11; 10 experimental and 1 control) was secured to the measurement platform using double-sided tape to avoid slippage. Care was taken to ensure the disk surface was level and in direct, parallel contact with the detector.

Measurement parameters followed the ISO 4287:1997 [27] Surface Texture: Profile Method standard with profile R, parameter 3 and Gauss filter. Cutoff lengths were 0.8 mm and 2.5 mm, the evaluation length was set at N = 5, pre/post-scan was ON, measuring speed was 0.5 mm/s, and auto range was turned on. The 2.5 mm cutoff was selected to match the specimen size, and measurements were repeated using the 0.8 mm cutoff to compare values. The measurement was repeated 3 times for each sample to create an average R_a_ roughness value. Data were recorded and exported to a spreadsheet for analysis.

### 2.3. Color and Translucency Measurements

Color and translucency were measured using a Konica Minolta CM-16d spectrophotometer (Konica Minolta Sensing, Osaka, Japan). This non-destructive test was performed on 11 specimens (10 experimental and 1 control), each 35 mm in diameter and 5 mm in thickness. Specimens were labeled with unique ID numbers on the side to avoid surface discoloration.

The spectrophotometer was connected to a laptop running SpectraMagic NX2 software (version 1.30.0006). After initializing the software, a dedicated folder was created for data collection. Calibration was performed using a zero-calibration box (CM-A182) (Konica Minolta Sensing, Osaka, Japan) and a white calibration cap, in accordance with manufacturer protocols. All calibrations were performed at room temperature to ensure consistent measurement conditions.

Specimens were positioned over the instrument port using a vertical leveling jig (CM-A304) (Konica Minolta Sensing, Osaka, Japan). Measurement settings included the following: observer angle 10°, illuminants D65 and F11, color space CIE L*a*b*, color difference equation ΔE*ab, automatic and manual averaging, and both SCI (specular component included) and SCE (specular component excluded) modes.

Color measurements as outlined in SO/TR 28642:2016 [28] Guidance on Color Measurement, were performed first, followed by translucency tests using the translucency parameter as defined by Johnston et al. [29]. Reflectance was compared over white and black backgrounds. A fully opaque sample yielded the same reflectance under both conditions, corresponding to 100% opacity, or 0% translucency. Each specimen’s CIE L*a*b* values were recorded. The color difference values from the baseline (control) sample were calculated using the following equation:

(1) $$\Delta E_{\left\{ab\right\}}=\;\sqrt{\left\{{\left(L_{2}^{*}\;-\;\;L_{1}^{*}\right)}^{2}\;+\;\;{\left(a_{2}^{*}\;-\;\;a_{1}^{*}\right)}^{2}\;+\;\;{\left(b_{2}^{*}\;-\;\;b_{1}^{*}\right)}^{2}\right\}}$$

where

L*_1_ is the L* value at T0

L*_2_ is the L* values at T1–10

a*_1_ is the a* value at T0

a*_2_ is the a* values at T1–10

b*_1_ is the b* value at T0

b*_2_ is the b* values at T1–10

Translucency parameter was measured as the difference between the CIE L* a* b* values with a white and black background using this formula:

(2) $$TP=\;\sqrt{\left\{{\left(L_{w}^{*}\;-\;\;L_{b}^{*}\right)}^{2}\;+\;\;{\left(a_{w}^{*}\;-\;\;a_{b}^{*}\right)}^{2}\;+\;\;{\left(b_{w}^{*}\;-\;\;b_{b}^{*}\right)}^{2}\right\}}$$

where

L*_w_ is the L* value measured on a white background

L*_b_ is the L* measured on a black background

a*_w_ is the a* measured on a white background

a*_b_ is the a* measured on a black background

b*_w_ is the b* measured on a white background

b*_b_ is the b* measured on a black background

### 2.4. Statistical Analysis

Statistical analysis was completed using statistical software (SPSS v29, IBM, Armonk, NY, USA). Descriptive statistics, including the minimum, maximum, means and standard deviation were calculated. Spearman’s ρ was calculated due to the small sample size and ordinal nature of the time data. A significance level of α = 0.05 was chosen.

## 3. Results

**Figure 1:**
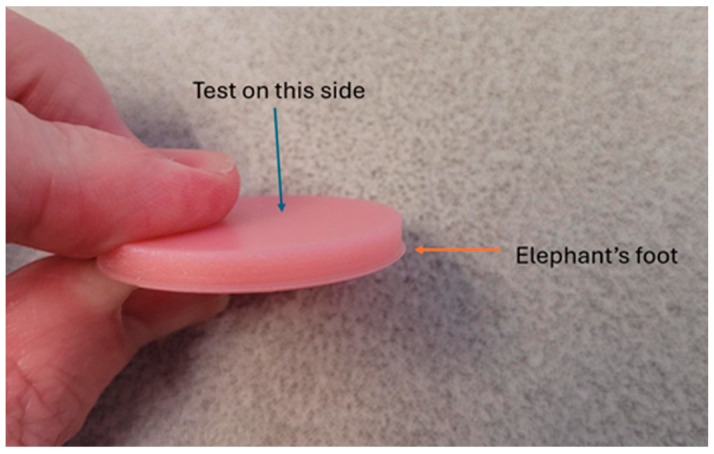
Printed specimen showing an “elephant’s foot” printing defect.

The addition of TiO_2_ to the denture base resin led to unacceptable increases in surface roughness, as well as color differences that were well outside of the accepted range of ΔE_ab_ < 3.7 [30]. Testing was performed on a surface distant to the print bed. This limited the effects of specimen damage that were produced during removal from the print bed, or from a defect such as “elephant’s foot” (Figure 1). Descriptive statistics are displayed in Table 1.

### 3.1. Printability of TiO_2_-Modified Denture Base Resin

The resin printed successfully at timepoints T1–T9, with all specimens achieving the proper dimensions. At T10, 8 weeks after mixing, one disk achieved the proper dimensions and one was approximately 0.5 mm thick. The remaining samples could not be printed.

### 3.2. Surface Roughness

**Figure 2:**
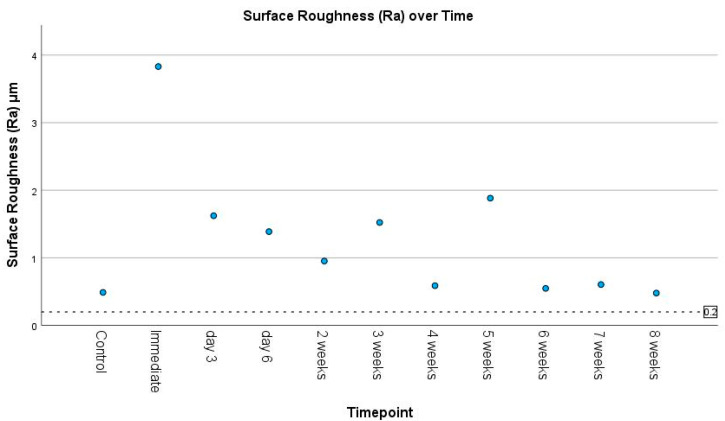
Scatterplot displaying the Surface roughness over time. R_a_ = 0.2 μm is the clinically acceptable limit, indicated by the dotted line.

Surface roughness was variable, as R_a_ ranged from a maximum value of 3.83 μm at T1, to a minimum value of 0.479 μm at T10. The mean surface roughness at T10 was similar to the control sample that was printed prior to adding TiO_2_ (R_a_ = 0.489, Figure 2).

### 3.3. Color Difference and Translucency

Translucency parameter (TP), ΔE_ab_ color difference, SCI L*, SCI a*, and SCI b* showed inconsistent decreases between time T0 and T10.

**Figure 3:**
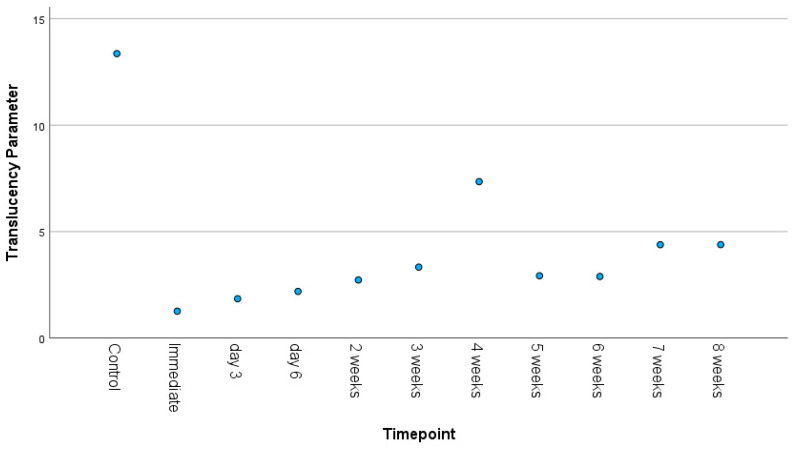
Scatterplot displaying translucency parameter over time.

The translucency parameter calculates the difference in light transmission for specimens measured on white and black backgrounds. A higher value indicates more translucency, or less opacity. Figure 3 presents translucency parameter values across time. Translucency was highest for the control samples (TP = 13.36) and lowest at T0 (TP = 1.26). Translucency gradually increased over time, except for a spike observed at week 4.

**Figure 4:**
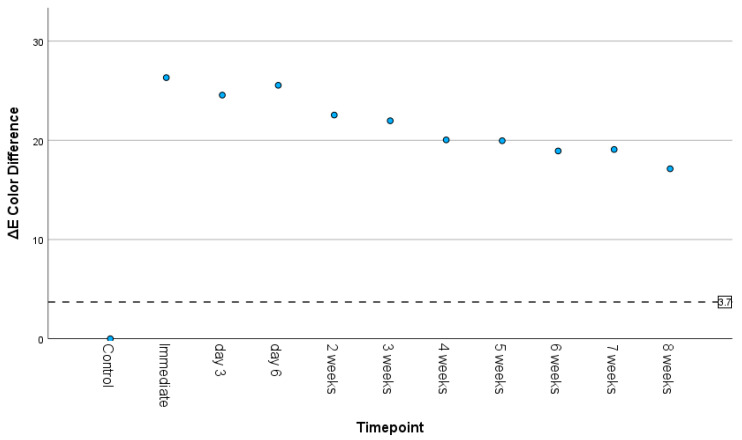
Scatterplot displaying the ΔE color difference over time. ΔE color difference of 3.7 is the clinically acceptable limit for color difference.

ΔE_ab_ color differences compared the experimental samples with the unmodified resin. Figure 4 displays color differences ranging from ΔE_ab_ = 0 for the control group to ΔE_ab_ = 26.32 for timepoint T1. ΔE* steadily drops to a low of ΔE_ab_ = 17.13 at T10. The reference line at ΔE_ab_ = 3.7 is the upper end of the reported acceptable value reported by Khashayar [30].

**Figure 5:**
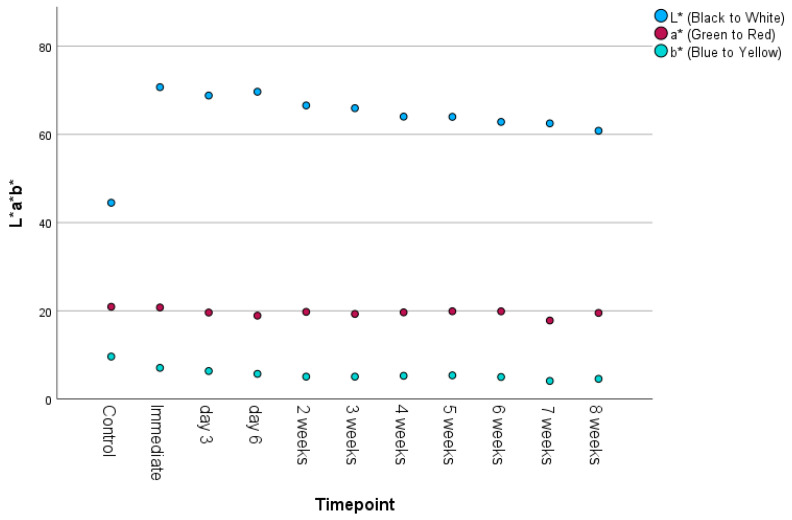
Scatterplot displaying the L*a*b* values over time.

Of the individual color parameters SCI L*, SCI a*, and SCI b*, only SCI L* showed remarkable color change over time (Figure 5). When added to the unmodified resin, the L* value increased nearly 30 units. Since the L* parameter is a measure of black to white, and the chosen additive was white in color, this change was expected. Over time L* gradually decreased, with the value being nearly 10 units lower at 8 weeks, which indicated specimen darkening. For SCI a*, the red-green parameter, little, if any change was noted following nanoparticle addition or across time. SCI b*, the blue-yellow parameter, decreased 2.7 units following TiO_2_ addition, indicating a slight loss of yellowness. However, little change was noted at subsequent timepoints.

### 3.4. Correlations Between Timepoint and Surface Roughness, Color Difference, and Translucency

Spearman’s ρ was calculated to determine the correlation between the time of printing and the tested properties. Table 2 contains the correlation coefficient and significance level for each correlation. SCI a* was not significant (ρ = −0.261 *p* = *0*.467). All remaining correlations were significant at the α = 0.05 level.

### 3.5. Correlations Between Surface Roughness and Color Difference, and Translucency

Spearman’s ρ was calculated to determine the correlation between surface roughness and the optical properties. Table 3 shows correlation coefficients and significance levels for each correlation. Except for SCI a* (ρ = 0.073, *p* = 0.832) and SCI b* (ρ = −0.419, *p* = 0.199), correlations were significant at the α = 0.05 level.

### 3.6. Limitations

This study was limited by its small sample size. A single sample from each timepoint was chosen for analysis due to only a single sample surviving at the final timepoint. The choice of one size of TiO_2_ nanoparticles (30 nm) limits the generalizability of this data, as multiple sizes of these nanoparticles are commercially available. No mechanical properties were tested, which would be required before a formulation could be approved for clinical use. The failures noted with this formulation may not apply to other formulations. Future research of different nanoparticle mixtures across time is warranted, and comparisons with prior research are needed.

## 4. Discussion

The aim of this study was to assess the printability and maintenance of surface properties over time in nano-TiO_2_-filled denture polymers. The null hypothesis, that surface roughness and optical properties would not change over time, was partially rejected. Surface roughness, translucency parameter, ΔE_ab_ color change and color parameter SCI L* underwent significant changes over an eight-week period. Color parameters SCI a* and SCI b* remained stable, without demonstrating significant change.

Color and translucency stability are important for a denture because it must maintain an adequate color range to match biological tissues. TiO_2_ was added because of its potential for reducing microbial activity that may occur when the denture contacts surrounding soft tissues. Here its addition significantly whitened the denture polymer, raising ΔE_ab_ values nearly 27 units. Denture color is influenced by pigment color, nanoparticle size and quantity, and the differences in refractive indices between polymer and nanoparticle. PMMA has a refractive index of 1.49, whereas TiO_2_ is more than 2.6. For materials with higher refractive indices, light travels shorter paths because it is bent more, and consequently penetrates less deeply. TiO_2_ effectively prevents light transmission and hence its scattering effect gives the material a whiter appearance. This explains higher SCI L* values, which in turn raise ΔE_ab_ values.

With later printings, this color discrepancy was partly recovered, with the ΔE_ab_ color change from baseline being reduced from 27 to 17 units. These results have two implications. The first is that printing may need to occur in two stages. The intaglio surfaces, where denture color is not visible, would benefit from TiO_2_ additions and hence require one printing stage. The remaining structure would be printed without TiO_2_ to preserve appropriate color matching with soft tissues. A second implication is that TiO_2_ particles likely migrate downward over time, accumulating near the bottom of the polymer. Although this offers better translucency, color matching and roughness properties at the top surface, it also implies that particle dispersion is non-uniform. This in turn may reduce any advantage rendered by the nanoparticles for improving bulk physical properties. Bulk properties were not measured in this study.

Denture smoothness is important because it offers both patient comfort and reduces plaque accumulation during wear. In traditional denture construction, the tissue surface is not polished, to maintain acceptable smoothness. The accepted roughness value to mitigate plaque accumulation is R_a_ < 0.2 μm [31]. The reference line shown near the bottom of Figure 3 is at this value. It is noteworthy that none of the printed samples were below this level. This suggests that for 3D-printed dentures, an alternative strategy is needed. The application of a coating or glaze would overcome the inherent roughness, since polishing the intaglio surface of a denture is normally avoided to prevent altering prosthesis fit. Compared to milled or traditionally manufactured materials, 3D-printed denture bases have been shown to possess higher surface roughness [32,33,34]. Glazes have been shown to improve color stability by decreasing surface roughness and covering surface defects that undergo staining, albeit temporarily [35,36].

**Figure 6:**
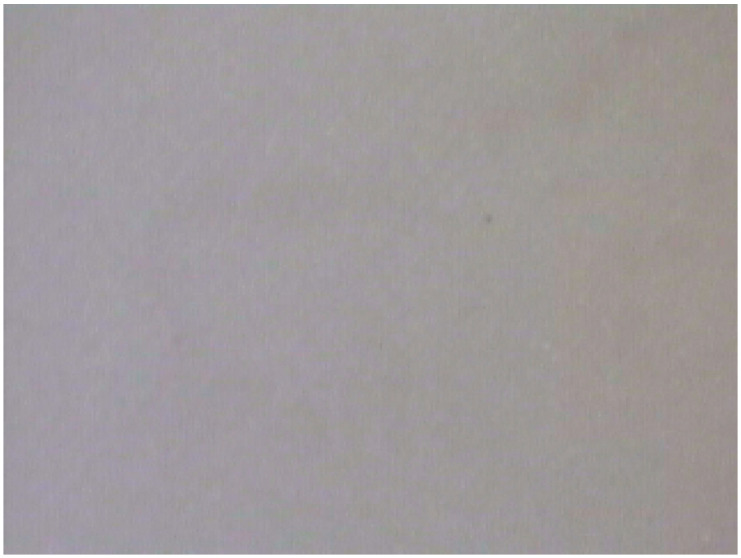
Sample at T3, at 30× magnification, showing a non-porous surface.

All prints were performed at 0 degrees, which has been shown by Shim et al. to yield lower surface roughness than either 45 degree or 90 degree print angles [37]. However, the same study also reported that more than twice as many samples printed at 0 degrees showed *C. albicans* overgrowth, as compared to those printed at 45 degrees or 90 degrees. This was attributed to pores being present across the surface of the 0° samples. Presumably this provided a substrate for fungal adherence. Porosity was not observed on the surfaces of the printed specimens in this study, as can be seen in Figure 6.

Recent studies have highlighted the variable impact nanoparticle incorporation renders on the surface properties of denture base resins. Alshaikh et al. [21] observed a small, statistically insignificant, increase in surface roughness with the addition of ZrO_2_ nanoparticles to a 3D-printed resin. AlGhamdi et al. [38] found that the effects of titanium nanoparticle incorporation were highly resin-dependent, with one formulation demonstrating a significant increase in surface roughness and unacceptable color change, while another resin showed minimal effects. A systematic review by Kaurani et al. [25] addresses the influence of nanoparticle size, reporting greater roughness with TiO_2_ particles smaller than 50 nm. This is attributed to the greater propensity for smaller particles to agglomerate, thereby creating greater surface area with irregularity. Collectively these findings align with those reported here, where incorporation of 30 nm TiO_2_ nanoparticles resulted in significantly elevated surface roughness and color difference with unmodified resin.

This preliminary study presents initial information on how the surface and optical properties of a TiO_2_-modified 3D-printed resin change over time, from initial mixing through print failure. Notably, surface roughness, translucency, and color difference were highest at T0 (immediately after mixing) and declined variably across eight weeks. A pronounced spike in translucency at week 4 (T5), paired with a drop in surface roughness, suggests uneven sedimentation or perhaps excessive agglomeration of nanoparticles occurred at this time period, which was possibly overcome during mixing at later periods. Although not measured here, it is expected that a non-uniform particle distribution would contribute to inconsistencies in optical and surface roughness properties. The printer used in this study included a built-in heater and mechanical mixing paddle to maintain resin homogeneity. However, these features may have been insufficient to prevent nanoparticle settling over time. Future printer designs should consider optimizing paddle geometry, mixing frequency, and thermal control to improve nanoparticle dispersion stability. The observed increase in surface roughness may also have accelerated degradation of the polydimethylsiloxane (PDMS) resin tray liner. This could potentially shorten the functional life of the printer tray, which is a concern for clinical users. This degradation was not noted visually during the trials.

Clinically, these findings highlight the challenges of incorporating TiO_2_ nanoparticles into printable denture base resins. At present, no commercially available denture base resin is validated for point-of-care 3D printing with embedded nanofillers. Users must strictly adhere to manufacturer instructions and avoid off-label modifications, as these may compromise material performance and device longevity. Furthermore, new surface-finishing strategies should be explored to mitigate roughness. Preliminary evidence supports the use of post-print glaze applications by Hashemzade [36] and Raszerski [35] to improve surface smoothness, although long-term clinical studies are needed to validate their effectiveness and durability.

## 5. Conclusions

The results of this in vitro study support the following conclusions.

1. TiO_2_ additions to a denture polymer produced significant changes in surface roughness and color that were not clinically acceptable.
2. Although printability of the resin–TiO_2_ mixture was maintained for 7 weeks, mixture homogeneity is questioned.
3. Dentures printed with TiO_2_ nanoparticles may require two-stage printing and a coating application.

## Figures and Tables

**Table 1:** Descriptive statistics for surface roughness (R_a_, µm), Translucency parameter, color difference (ΔE_ab_), and CIE Lab* values (SCI Mode) **.

Descriptive Statistics	N	Minimum	Maximum	Mean	Std. Deviation
Surface Roughness (R_a_, μm)	11	0.479	3.83	1.264	0.995
Translucency Parameter	11	1.26	13.36	4.242	3.439
ΔE_ab_ Color Difference	11	0	26.32	19.640	7.145
SCI L*	11	44.5	70.69	63.658	7.091
SCI a*	11	17.81	20.92	19.646	0.840
SCI b*	11	4.12	9.64	5.76	1.518

** Note: SCI L*, a* and b* values represent white-black, red-green, and yellow-blue color dimensions, respectively, as measured in specular component included (SCI) mode, using a reflectance spectrophotometer.

**Table 2:** Correlation to timepoint (Spearman’s rho).

Correlation to Timepoint	Spearman’s ρ	Significance (2-Tailed)
Surface Roughness	−0.733	0.016 *
Translucency Parameter	0.806	0.005 *
ΔE_ab_ color shift	−0.976	<0.001 *
SCI L*	−0.988	<0.001 *
SCI a*	−0.261	0.467
SCI b*	−0.875	<0.001 *

* indicates statistical significance.

**Table 3:** Correlation to surface roughness (Spearman’s rho).

Correlation to Roughness	Spearman’s ρ	Significance (2-Tailed)
Translucency Parameter	−0.727	0.011 *
ΔE_ab_ color shift	0.791	0.004 *
SCI L*	0.773	0.005 *
SCI a*	0.073	0.832
SCI b*	0.419	0.199 *

* indicates statistical significance.

## Data Availability

The raw data supporting the conclusions of this article will be made available by the authors on request.

## Abbreviations

The following abbreviations are used in this manuscript:

TiO_2_	Titanium dioxide
SLA	Stereolithography
R_a_	Roughness Average
STL	Standard Tessellation Language
CIE Lab*	Commission Internationale de l’Éclairage color space
